# Performance Assessment of Smartphone Tightly Coupled PPP/INS Integration with an Adaptive Robust Kalman Filter

**DOI:** 10.3390/s26175320

**Published:** 2026-08-22

**Authors:** Hongyu Zhu, Haiping Xiao, Zhiqiang Li, Xinqian Guan, Jianfan Lai

**Affiliations:** 1 School of Airspace Technology, Jiangxi University of Science and Technology, Ganzhou 341000, China; hpxiao@jxust.edu.cn (H.X.); zhiqiangli@jxust.edu.cn (Z.L.); guanxingian@mail.jxust.edu.cn (X.G.); laijianfan@mail.jxust.edu.cn (J.L.); 2 Key Laboratory of Low Dimensional Quantum Materials and Sensor Devices of Jiangxi Education Institutes, Jiangxi University of Science and Technology, Ganzhou 341000, China

**Keywords:** adaptive robust Kalman filter, tightly coupled integration, PPP/INS, smartphone positioning, complex urban environment

## Abstract

**Highlights:**

**What are the main findings?**

- A comprehensive tightly coupled PPP/INS framework tailored for smartphones is developed, leveraging raw measurements from the integrated GNSS and IMU chipsets of smartphones.
- An adaptive factor adjustment method based on dynamic intensity and geometric precision indicators is proposed, constructing a robust adaptive Kalman filtering model that integrates the IGG-III weight function.

**What are the implications of the main findings?**

- This study validates the effectiveness of the adjustment mechanism combining robust estimation and adaptive factor in the tightly coupled integration of low-cost MEMS-IMU and smartphone GNSS, and provides technical support for high-precision navigation of intelligent mobile terminals in complex urban environments.
- The proposed ARKF-based tightly coupled framework is suitable for scenarios with strict requirements for positioning continuity and reliability, such as intelligent transportation systems, location-based services, and autonomous driving. It can provide a technical reference for realizing high-precision positioning with consumer-grade terminals.

**Abstract:**

To address the challenges of GNSS signal blockages and severe multipath effects in complex urban environments, this paper proposes a tightly coupled precise point positioning (PPP)/inertial navigation system (INS) integration method based on an adaptive robust Kalman filter (ARKF) for smartphones. The proposed method integrates a robust estimation module based on the IGG-III weight function and an adaptive factor derived from vehicle dynamic intensity and geometric precision indicators, to mitigate observation outliers and dynamic model errors. To evaluate the positioning performance of this algorithm, two typical vehicle experiments based on the GNSS and inertial measurement unit (IMU) chipsets of the Xiaomi Mi 8, as well as an external H30 IMU, were conducted. Experimental results show that in the urban expressway environment, the horizontal root mean square (RMS) error of the loosely coupled PPP/INS solution was reduced by 74.17% compared with the conventional PPP solution, while the maximum horizontal positioning error of the tightly coupled PPP/INS solution was reduced by 44.82% compared with the loosely coupled PPP/INS solution. In the complex urban road and tunnel environments, the proposed ARKF-based tightly coupled PPP/INS method achieved a 36.79% reduction in horizontal RMS error compared with the tightly coupled PPP/INS solution based on the standard extended Kalman filter (EKF) and demonstrated more robust positioning performance in the tunnel.

## 1. Introduction

In 2016, the release of Android Nougat opened access to raw global navigation satellite system (GNSS) measurements, which sparked widespread interest and research in smartphone precise point positioning (PPP) [1]. However, due to the limitations of consumer-grade hardware, the measurements from low-cost GNSS chipsets in smartphones suffer from significant noise and biases, resulting in unreliable observations. Furthermore, the GNSS positioning performance is severely affected by blockages and multipath effects in challenging environments such as urban canyons [2]. Fortunately, smartphones incorporate inertial measurement unit (IMU) chipsets that can independently provide continuous and high-frequency navigation data when the GNSS signals are temporarily unavailable. Although the loosely coupled (LC) and tightly coupled (TC) solutions are widely employed for GNSS/ inertial navigation system (INS) integration in high-precision geodetic receivers, it remains unclear whether these solutions can be effectively applied to consumer-grade smartphones. In particular, tightly coupled PPP/INS integration that relies on raw GNSS observations is still in the exploratory stage.

Research on GNSS and INS integration using smartphones has been conducted for over a decade [3,4]. Early research primarily employed iPhones to collect IMU sensor data, integrating these measurements with observations from an external GPS receiver to validate the feasibility of smartphone-based gyroscope and accelerometer measurements for navigation under dynamic conditions [5]. In May 2018, the Xiaomi Mi 8 smartphone equipped with the Broadcom BCM47755 dual-frequency GNSS chipset was released, which significantly promoted the development of dual-frequency PPP models using smartphones [6,7,8]. Among these models, the uncombined observation approach offers an advantage over the ionosphere-free combination by using single- and dual-frequency observations. This is particularly beneficial for positioning with the embedded passive linearly polarized GNSS antennas of smartphones in urban environments, as it enhances the satellite geometry and observation redundancy [9]. However, compared with geodetic receivers equipped with right-hand circularly polarized (RHCP) antennas, the linearly polarized antennas of smartphones are almost unable to suppress multipath, resulting in a significant reduction in GNSS signal strength in areas with slight obstructions, and causing frequent carrier phase cycle slips and even loss of lock. Meanwhile, polarization mismatch results in a signal power loss of approximately 3 dB-Hz [10]. Currently, researchers have enhanced the PPP performance of smartphones through a series of improvement strategies, including signal-to-noise-ratio-based stochastic model reconstruction, independent clock parameterization, atmospheric augmentation, and slant ionospheric constraints [8,11,12,13].

In parallel, some studies have investigated GNSS/INS integrated navigation technology by using data from the IMU embedded in smartphones [14]. Yan et al. [15] processed the IMU data from the Mi 8 and Honor Play smartphones using a 15-state Kalman filter for a GNSS/INS coupled navigation model, demonstrating that the solutions derived from smartphone IMUs were comparable to those from a higher-grade reference IMU, with errors of approximately 0.2 m in position and 1 degree both in roll and pitch, whereas the yaw error was larger, reaching several degrees. Yi et al. [9] proposed a method that integrated INS and single- and dual-frequency PPP models based on low-cost smartphone GNSS/IMU chipsets, achieving roughly 2 m horizontal accuracy in urban environments. Yang et al. [16] proposed a tightly coupled smartphone PPP/INS method that incorporated global ionospheric map (GIM) constraints, and this approach achieved a horizontal accuracy of 1.6 m in open-sky conditions and reduced horizontal errors by 80% in GNSS-challenged environments. Yi et al. [17] introduced the Galileo high accuracy service (HAS) into the smartphone PPP/INS integrated model and achieved navigation and positioning in urban environments using broadcast ephemeris.

The Kalman filter (KF) is a commonly used fusion algorithm in GNSS/INS integrated navigation systems [18]. Pourmina et al. [19] developed an RTK/INS integration method based on the extended Kalman filter (EKF) for smartphones, achieving horizontal positioning accuracies of approximately 0.380 m and 0.415 m for the Xiaomi Mi 8 and Samsung Galaxy S21 Ultra, respectively, in complex urban environments. Elkhalea et al. [20] proposed a smartphone algorithm that combines GNSS with a modified downdate algorithm (MDDA) for satellite selection and integrates INS in both loosely and tightly coupled configurations using an extended Kalman filter. Chen et al. [21] proposed a variational GNSS/INS robust filter based on an improved Student’s t model, incorporating a resilient information transfer strategy and an adaptive forgetting factor to improve robustness against measurement noise. Ma et al. [22] proposed an improved multiple-outlier-robust Kalman filter (MORKF) algorithm to enhance the accuracy and robustness of tightly coupled GNSS/INS positioning. The existing adaptive robust algorithms usually rely on adaptive statistical methods based on innovations or residuals, and have been validated mostly on geodetic-grade receivers. However, these algorithms have not been sufficiently tailored to the distinct error characteristics of smartphone GNSS antennas and micro-electro-mechanical system (MEMS) IMUs.

The purpose of this paper is to systematically assess the performance of a tightly coupled PPP/INS integration with the proposed adaptive robust Kalman filter for smartphones in complex urban environments. The main contributions are summarized as follows.

(1) A tightly coupled uncombined PPP/INS model is constructed based on GNSS observations and IMU measurements from smartphones. The SIGMA-Δ stochastic model considering satellite signal diffraction is introduced, and a tight integration of single- and dual-frequency multi-GNSS observations with IMU measurements is established. This can effectively constrain the divergence of INS errors even when only a single satellite is visible.
(2) An improved adaptive factor adjustment method is proposed, which introduces accelerometer measurement variance and position dilution of precision (PDOP) as external indicators to adjust the adaptive factor thresholds. The method directly utilizes vehicle dynamics and satellite geometry information, enabling a threshold adjustment mechanism that enhances robustness against dynamic model errors in challenging urban environments.
(3) Two vehicle experiments are designed to evaluate the positioning performance of the proposed method. The results show that the proposed ARKF-based tightly coupled PPP/INS method achieves significantly higher positioning accuracy than EKF-based and robust Kalman filter (RKF)-based tightly coupled PPP/INS solutions in complex urban environments, and exhibits more stable positioning performance in the tunnel scenarios.

The remainder of this paper is structured as follows. Section 2 presents the mathematical model of tightly coupled PPP/INS integration. Section 3 establishes the tightly coupled PPP/INS integration scheme with the proposed adaptive robust Kalman filter. Section 4 presents the results and analysis of two vehicle experiments conducted in urban environments. Finally, Section 5 concludes the paper.

## 2. Tightly Coupled PPP/INS Mathematical Model

### 2.1. INS Error Model and Initialization

**Figure 1:**
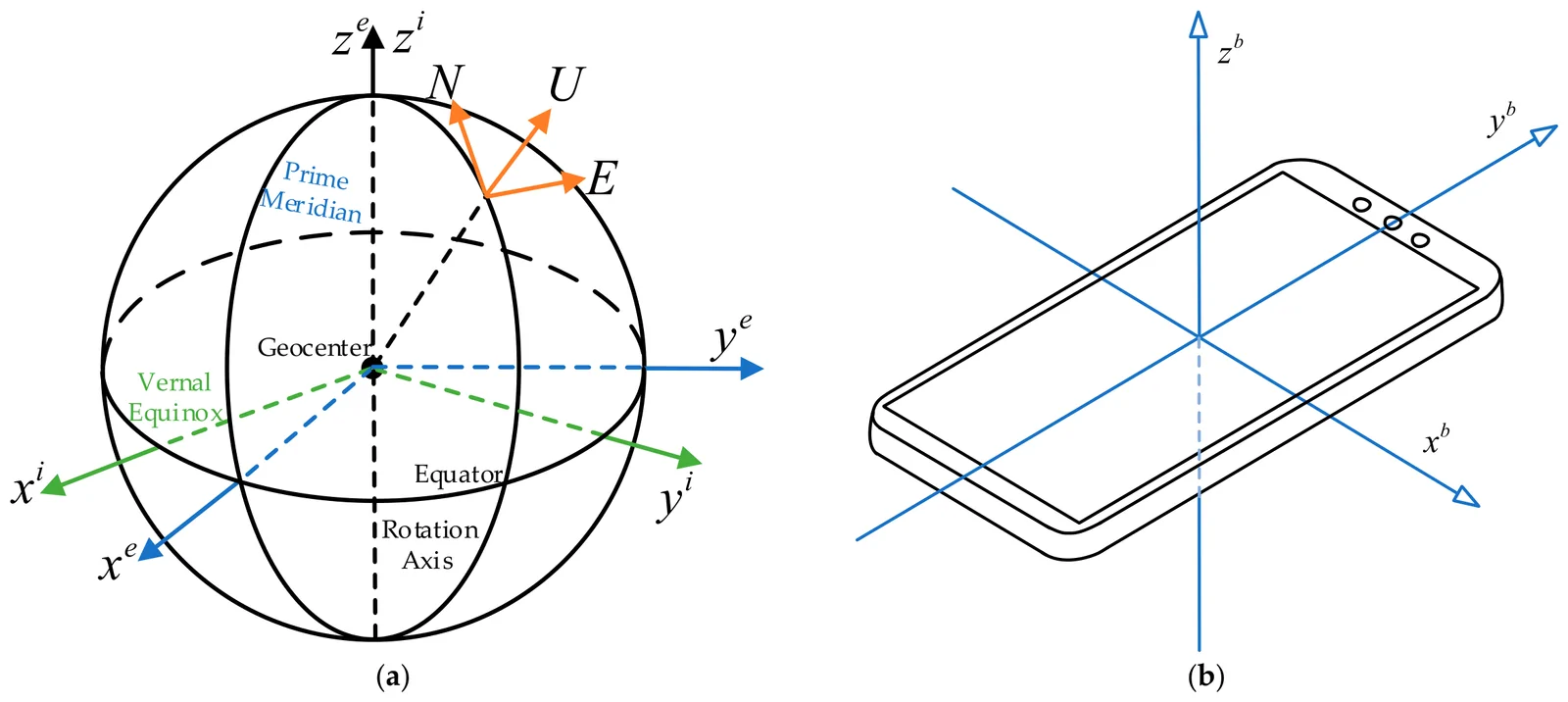
Common coordinate frames in inertial navigation. (**a**) Inertial frame (shown in green); Earth-centered, Earth-fixed frame (shown in blue); and navigation frame (shown in orange). (**b**) Smartphone body frame.

The commonly used coordinate systems in inertial navigation mainly include the inertial frame (*i*-frame); the Earth-centered, Earth-fixed frame (*e*-frame); the body frame (*b*-frame); and the navigation frame (*n*-frame), as shown in Figure 1. The smartphone *b*-frame follows the right-forward-up (RFU) convention, with its origin at the IMU measurement center. The *x*-axis points to the right, the *y*-axis points forward, and the *z*-axis points upward. The *n*-frame is typically defined as a local coordinate system, which is used to describe the kinematic state of the vehicle [23,24]. The angular velocity measured by the gyroscope is used to update the attitude matrix of the vehicle relative to the *n*-frame. Therefore, a coordinate transformation from the *n*-frame to the *e*-frame is required for data fusion with GNSS solutions expressed in the *e*-frame.

Based on the INS dynamic equations, the error state model for the inertial navigation system is expressed as follows [25]:

(1) $$\left(\begin{array}{c} \delta {\dot{r}}_{\mathrm{INS}}^{e}\\ \delta {\dot{v}}_{\mathrm{INS}}^{e}\\ {\dot{\varphi }}^{e} \end{array}\right)=\left(\begin{array}{c} \delta v_{\mathrm{INS}}^{e}+\xi_{r}\\ -2\Omega_{ie}^{e}\delta v_{\mathrm{INS}}^{e}+\left[\left(C_{b}^{e}f_{ib}^{b}\right)\times \right]\varphi^{e}-C_{b}^{e}\delta f_{ib}^{b}+\xi_{v}\\ -\Omega_{ie}^{e}\varphi^{e}+C_{b}^{e}\delta \omega_{ib}^{b}+\xi_{\varphi } \end{array}\right)$$

where $\delta r_{\mathrm{INS}}^{e}$, $\delta v_{\mathrm{INS}}^{e}$, and $\varphi^{e}$ are the position, velocity, and misalignment error vectors in the *e*-frame, respectively; a dot over a symbol denotes its time derivative. The process noise vectors $\xi_{r}$, $\xi_{\nu }$, and $\xi_{\varphi }$ corresponding to the position, velocity, and attitude states are modeled as independent zero-mean Gaussian white noise. $\Omega_{ie}^{e}$ is the skew-symmetric matrix of the earth rotation rate vector $\omega_{ie}^{e}$; $C_{b}^{e}$ is the rotation matrix from the *b*-frame to the *e*-frame; $f_{ib}^{b}$ and $\delta f_{ib}^{b}$ are the specific force vector and its error, respectively; $\left[\left(C_{b}^{e}f_{ib}^{b}\right)\times \right]$ is the skew-symmetric matrix form of $\left(C_{b}^{e}f_{ib}^{b}\right)$; and $\delta \omega_{ib}^{b}$ is the error of gyroscope angular velocity.

The compensation for the biases of the accelerometer and gyroscope, neglecting scale factor and cross-coupling errors, is modeled as:

(2) $$\delta f_{ib}^{b}=\delta b_{a}+\varepsilon_{a}$$

(3) $$\delta \omega_{ib}^{b}=\delta b_{g}+\varepsilon_{g}$$

where $\delta b_{a}$ and $\delta b_{g}$ denote the bias vectors of the accelerometer and gyroscope, respectively; $\varepsilon_{a}$ and $\varepsilon_{g}$ are the corresponding process noise vectors, modeled as zero-mean Gaussian white noise. Following the INS error-state dynamics, the 15-dimensional error state vector is defined, including position, velocity, attitude, and the biases of the accelerometer and gyroscope:

(4) $$X_{INS}={\left[\begin{array}{ccccc} \delta r_{\mathrm{INS}}^{e}, & \delta v_{\mathrm{INS}}^{e}, & \varphi^{e}, & \delta b_{a}, & \delta b_{g} \end{array}\right]}^{T}$$

For integrated navigation with low-cost MEMS IMUs, a common method is to estimate the yaw angle using the forward velocity provided by GNSS [26]. This velocity needs to exceed 2.0 m/s, as the accuracy of the yaw estimate degrades significantly at lower speeds [27]. When the forward axis of the vehicle is aligned with its velocity vector, the roll angle is initialized to $0^{\circ }$ with an uncertainty of ${\pm 5}^{\circ }$, while the pitch and heading angles are initialized using GNSS-derived position and velocity solutions [15]:

(5) $$\left\{\begin{array}{c} \theta =\arctan\left(\nu_{U,GNSS}/\sqrt{v_{N,GNSS}^{2}+v_{E,GNSS}^{2}}\right)\\ \varphi =0^{\circ }\pm \Delta \varphi \\ \psi =\arctan\left(v_{E,GNSS}/v_{N,GNSS}\right) \end{array}\right.$$

where θ, φ, and ψ are the pitch, roll, and yaw angles, respectively; ν is the velocity vector in the *n*-frame; E, N, and U denote east, north, and up, respectively; Δφ is the uncertainty. Furthermore, INS navigation states are computed with reference to the IMU measurement center, while GNSS measurements are referenced to the antenna phase center. This spatial deviation must be corrected to align all data to a common reference point on the platform. The position and velocity can be transformed using the following expressions:

(6) $$r_{\mathrm{GNSS}}^{e}=r_{\mathrm{INS}}^{e}+C_{b}^{e}l^{b}$$

(7) $$v_{\mathrm{GNSS}}^{e}=v_{\mathrm{INS}}^{e}+C_{b}^{e}\Omega_{ib}^{b}l^{b}-\Omega_{ie}^{e}C_{b}^{e}l^{b}$$

where $l^{b}$ is the lever arm correction vector in the body frame; $\Omega_{ib}^{b}$ is the skew-symmetric matrix of the gyroscope angular velocity $\omega_{ib}^{b}$.

### 2.2. Tightly Coupled PPP/INS Measurement Equation

The tightly coupled PPP/INS solution based on raw measurements calculates predicted pseudorange and carrier phase observation values for each visible satellite using INS-derived navigation states. The advantage of this approach is that it can constrain and correct INS errors even when only one satellite observation is available, thereby achieving tighter integration and higher availability. For a tightly coupled PPP/INS model integrating observations of GPS, BDS, and GLONASS, the linearized measurement equation is formulated as:

(8) $$Z_{\kappa }=H_{\kappa }X_{PPP/INS}+\varepsilon_{\kappa }$$

where $Z_{\kappa }$ is the innovation vector, defined as the difference between the raw GNSS observations and the INS-predicted measurements; $\varepsilon_{\kappa }$ is the observation noise vector, which includes multipath effects, measurement noise, and other unmodeled errors. $H_{\kappa }$ is the design matrix corresponding to the state vector $X_{PPP/INS}$, and its specific structure is given by:

(9) $$H_{\kappa }=\left[\begin{array}{cc} H_{INS,P}^{s,f} & H_{GNSS,P}^{s,f}\\ H_{INS,L}^{s,f} & H_{GNSS,L}^{s,f} \end{array}\right]$$

where $H_{INS,P}^{s,f}$ and $H_{INS,L}^{s,f}$ can be written as:

(10) $$H_{INS,P}^{s,f}=\left[\begin{array}{ccccc} H_{P}^{G,f_{1}} & 0 & H_{A}^{G,f_{1}} & 0 & 0\\ H_{P}^{G,f_{5}} & 0 & H_{A}^{G,f_{5}} & 0 & 0\\ H_{P}^{B,f_{1}} & 0 & H_{A}^{G,f_{1}} & 0 & 0\\ H_{P}^{R,f_{1}} & 0 & H_{A}^{G,f_{1}} & 0 & 0 \end{array}\right]$$

(11) $$H_{INS,L}^{s,f}=\left[\begin{array}{ccccc} H_{L}^{G,f_{1}} & 0 & H_{A}^{G,f_{1}} & 0 & 0\\ H_{L}^{G,f_{5}} & 0 & H_{A}^{G,f_{5}} & 0 & 0\\ H_{L}^{B,f_{1}} & 0 & H_{A}^{G,f_{1}} & 0 & 0\\ H_{L}^{R,f_{1}} & 0 & H_{A}^{G,f_{1}} & 0 & 0 \end{array}\right]$$

where the superscript G denotes GPS; $H_{P}^{S,f_{1}}$ is the unit line-of-sight vector from the INS-predicted position to the satellite; $H_{A}^{s}$ is the coefficient matrix for the attitude angles. $H_{GNSS,P}^{s,f}$ and $H_{GNSS,L}^{s,f}$ can be expressed as:

(12) $$H_{GNSS,P}^{s,f}=\left[\begin{array}{ccccccccccc} 1 & 0 & 0 & M_{w}^{G} & H_{I}^{G,f_{1}} & 0 & 0 & 0 & 0 & 0 & 0\\ 1 & 0 & 0 & M_{w}^{G} & H_{I}^{G,f_{5}} & 0 & 0 & 0 & 0 & 0 & 0\\ 1 & 1 & 0 & M_{w}^{B} & 0 & H_{I}^{B,f_{1}} & 0 & 0 & 0 & 0 & 0\\ 1 & 0 & 1 & M_{w}^{R} & 0 & 0 & H_{I}^{R,f_{1}} & 0 & 0 & 0 & 0 \end{array}\right]$$

(13) $$H_{GNSS,L}^{s,f}=\left[\begin{array}{ccccccccccc} 1 & 0 & 0 & M_{w}^{G} & -H_{I}^{G,f_{1}} & 0 & 0 & H_{N_{1}}^{G,f_{1}} & 0 & 0 & 0\\ 1 & 0 & 0 & M_{w}^{G} & -H_{I}^{G,f_{5}} & 0 & 0 & 0 & H_{N_{5}}^{G,f_{5}} & 0 & 0\\ 1 & 1 & 0 & M_{w}^{B} & 0 & -H_{I}^{B,f_{1}} & 0 & 0 & 0 & H_{N_{1}}^{B,f_{1}} & 0\\ 1 & 0 & 1 & M_{w}^{R} & 0 & 0 & -H_{I}^{R,f_{1}} & 0 & 0 & 0 & H_{N_{1}}^{R,f_{1}} \end{array}\right]$$

where $M_{w}^{s}$ is the tropospheric mapping function matrix; $H_{I}^{s}$ is the coefficient matrix for the ionospheric delay parameters; and $H_{N}^{s}$ denotes the coefficient matrix for ambiguity parameters. $X_{PPP/INS}$ is the state vector, which is constructed with the estimated parameters from the uncombined PPP model.

(14) $$X_{PPP/INS}={\left[\begin{array}{cccccccc} \delta r_{\mathrm{INS}}^{e}, & \delta v_{\mathrm{INS}}^{e}, & \varphi^{e}, & \delta b_{a}, & \delta b_{g}, & cdt_{r}, & ISB^{B}, & ISB^{R},\\ T, & {\hat{I}}_{1}^{G}, & {\hat{I}}_{1}^{B}, & {\hat{I}}_{1}^{R}, & {\hat{N}}_{1}^{G}, & {\hat{N}}_{5}^{G}, & {\hat{N}}_{1}^{B}, & {\hat{N}}_{1}^{R} \end{array}\right]}^{T}$$

where the superscripts G, B, and R denote the GPS, BDS, and GLONASS systems, respectively; c is the speed of light; $dt_{r}$ denotes receiver clock error; ${ISB}^{s}$ is the inter-system bias relative to GPS; T is the tropospheric delay; I is the ionospheric delay; and N is the ambiguity parameter. This uncombined model with single- and dual-frequency multi-GNSS observations enhances the availability and redundancy of the system, but it also significantly increases the number of state parameters, increases the dimension of the state covariance matrix during the Kalman filter time update step, and may reduce computational efficiency.

## 3. Tightly Coupled PPP/INS Integration with ARKF

### 3.1. Stochastic SIGMA-Δ Model

For low-cost GNSS receivers, the construction of the stochastic model will directly affect the accuracy of parameter estimation during the positioning process [28]. This study is based on the SIGMA-Δ model [29] and accounts for signal diffraction errors [30]. The variance model of the GNSS observations is constructed as follows:

(15) $$\sigma_{\Delta }^{2}(C/N_{0})=C_{S}\cdot 10^{-({C/N_{0}}_{\mathrm{measured}}-{C/N_{0}}_{\mathrm{template}})/10}$$

and

(16) $$C_{S}=B_{S}{\left[\lambda /2\pi \right]}^{2}$$

where $C_{S}$ is a constant derived from the carrier tracking loop bandwidth $B_{S}$; the C/N_0_ template is defined as the C/N_0_ reference value, which can be set to 40 dB/Hz [12].

### 3.2. Extended Kalman Filter Recursive Estimation

The Extended Kalman Filter is a standard method for state estimation in nonlinear systems. It linearizes the nonlinear system and measurement models through first-order Taylor series expansion around the current estimate, and performs recursive prediction and update steps [31,32]. Assume that the system state and observation equations are given as follows:

(17) $$x_{k+1}=f_{k}\left(x_{k}\right)+w_{k}$$

(18) $$z_{k}=h_{k}\left(x_{k}\right)+v_{k}$$

where $f\left(x_{k}\right)$ and $h\left(x_{k}\right)$ represent the nonlinear state transition and observation functions, respectively; $x_{k}$ is the state vector; $w_{k}$ and $v_{k}$ denote the process and measurement noise vectors. The initial state estimate ${\hat{x}}_{0}^{-}$ and its error covariance matrix $P_{0}^{-}$ are given by:

(19) $${\hat{x}}_{0}^{-}=E\left(x_{0}\right)$$

(20) $$P_{0}^{-}=E\left[\left(x_{0}-{\hat{x}}_{0}\right){\left(x_{0}-{\hat{x}}_{0}\right)}^{T}\right]$$

and the Kalman gain matrix is then computed using the state covariance and the measurement noise covariance:

(21) $$K_{k}=P_{k}^{-}H_{k}^{T}{\left[H_{k}P_{k}^{-}H_{k}^{T}+R_{k}\right]}^{-1}$$

where $H_{k}$ is the Jacobian matrix of the observation function, linearized at the prior state estimate ${\hat{x}}_{k}^{-}$.

(22) $$H_{k}\approx {\left.\frac{\partial h_{k}}{\partial x}\right|}_{{\hat{x}}_{k}^{-}}$$

Using the prediction error vector and the Kalman gain matrix, the prior state estimate ${\hat{x}}_{k}^{-}$ is corrected to obtain the posterior state estimate ${\hat{x}}_{k}$.

(23) $${\hat{x}}_{k}={\hat{x}}_{k}^{-}+K_{k}\left[z_{k}-h_{k}\left({\hat{x}}_{k}^{-}\right)\right]$$

Update error covariance:

(24) $$P_{k}=\left[I-K_{k}H_{k}\right]P_{k}^{-}$$

Predict the new state vector and state covariance matrix:

(25) $${\hat{x}}_{k}^{-}=f_{k-1}\left({\hat{x}}_{k-1},u_{k-1}\right)$$

(26) $$P_{k+1}^{-}=F_{k}P_{k}F_{k}^{T}+Q_{k}$$

where $F_{k}$ is the Jacobian matrix of the state transition function, linearized at the prior state estimate ${\hat{x}}_{k}^{-}$.

(27) $$F_{k}\approx {\left.\frac{\partial f_{k}}{\partial x}\right|}_{{\hat{x}}_{k}^{-}}$$

The EKF integrates the dynamic model of INS with the observation model of GNSS through a recursive predict-update framework. The INS propagates high-rate navigation states using its raw measurements during the prediction step, while GNSS observations are used to constrain and correct accumulated INS errors in the update step.

### 3.3. Adaptive Robust Kalman Filter Based on Dynamic Intensity and PDOP

The GNSS signals are susceptible to multipath and non-line-of-sight (NLOS) effects in urban environments, which leads to deviations in state estimation. Based on the principle of M-estimation, the adverse impact of abnormal observations can be mitigated by inflating the corresponding elements in the measurement noise covariance matrix R. Specifically, the weights are assigned to the observations based on the standardized innovation (prediction residuals) of the observations, thereby constructing an equivalent weight matrix $R^{*}$.

(28) $$R_{k}^{*}=R_{k}/\gamma_{i}$$

(29) $$\gamma_{i}=\left\{\begin{array}{cc} 1 & \left|{\bar{v}}_{i}\right|\leq d_{0}\\ \frac{d_{0}}{\left|{\bar{v}}_{i}\right|}{\left(\frac{d_{1}-\left|{\bar{v}}_{i}\right|}{d_{1}-d_{0}}\right)}^{2} & d_{0}<\left|{\bar{v}}_{i}\right|\leq d_{1}\\ 10^{-3} & \left|{\bar{v}}_{i}\right|>d_{1} \end{array}\right.$$

where $\gamma_{i}$ is the robust weight factor computed from the normalized innovation based on the IGG-III weight function [33,34]; $d_{0}$ and $d_{1}$ are threshold parameters, typically set within the ranges $d_{0}=1.0∼3.0$ and $d_{1}=3.0∼8.0$; ${\bar{v}}_{i}$ is the standardized residual [35].

(30) $${\bar{v}}_{i}=\frac{v_{i}}{\sqrt{{\hat{\sigma }}_{0}^{2}Q_{v_{i}}}}$$

(31) $${\hat{\sigma }}_{0}^{2}=\frac{\xi^{T}Q_{\xi }^{-1}\xi }{n}$$

where $v_{i}$ and $Q_{v_{i}}$ denote the observations residual and the corresponding variance, respectively; ξ is predicted residual vector, defined as $\xi =z_{k}-h_{k}\left({\bar{x}}_{k}\right)$, and $Q_{\xi }$ is the corresponding covariance matrix.

The INS dynamic model and IMU measurements may not accurately describe actual vehicle motion when vehicle dynamics change abruptly. Therefore, it is difficult for the predefined constant process noise covariance matrix to accurately reflect the instantaneous increase in model uncertainty, resulting in a significant discrepancy between the actual and presumed statistical characteristics of the model errors. The adaptive robust Kalman gain incorporating both an adaptive factor and a robust equivalent weight matrix is expressed as:

(32) $$K_{k}^{*}=\frac{1}{\alpha_{k}}P_{k}^{-}H_{k}^{T}{\left[\frac{1}{\alpha_{k}}H_{k}P_{k}^{-}H_{k}+R_{k}^{*}\right]}^{-1}$$

where $\alpha_{k}$ is the adaptive factor determined by the state discrepancy statistic and is commonly expressed as a three-segment function [36,37]. To further enhance the ability of the adaptive factor to reflect the instantaneous reliability of the system, this study proposes an adaptive threshold adjustment method that integrates the variance of accelerometer measurements with the PDOP as follows:

(33) $$\alpha_{k}=\left\{\begin{array}{ll} 1 & \left|\Delta {\tilde{x}}_{k}\right|\leq c_{0}^{*}\left(k\right)\\ \frac{\left|\Delta {\tilde{x}}_{k}\right|}{c_{0}^{*}\left(k\right)}{\left[\frac{c_{1}^{*}\left(k\right)-\left|\Delta {\tilde{x}}_{k}\right|}{c_{1}^{*}\left(k\right)-c_{0}^{*}\left(k\right)}\right]}^{2} & c_{0}^{*}\left(k\right)<\left|\Delta {\tilde{x}}_{k}\right|\leq c_{1}^{*}\left(k\right)\\ 10^{-3} & \left|\Delta {\tilde{x}}_{k}\right|>c_{1}^{*}\left(k\right) \end{array}\right.$$

where the state discrepancy statistic for model errors can be constructed as [38]:

(34) $$\Delta {\tilde{x}}_{k}=\frac{\left\|{\tilde{x}}_{k}-{\hat{x}}_{k}^{-}\right\|}{\sqrt{tr(P_{{\bar{x}}_{k}})}}$$

where tr denotes the matrix trace. The adaptive factor is constructed by the discrepancy between the predicted state from the model and the estimated state from the measurements. This statistic characterizes the relative mismatch degree of the system dynamic model. The square root of the predicted state covariance trace serves as a global scale metric of total state uncertainty, and quantifies the overall deviation of the state vector relative to the theoretical prediction uncertainty. The position and velocity states of the INS subsystem are adopted in the actual implementation. The norm of the discrepancy between the updated state estimate ${\tilde{x}}_{k}$ and the predicted state estimate ${\hat{x}}_{k}^{-}$ is given as [39]:

(35) $$\left\|{\tilde{x}}_{k}-{\hat{x}}_{k}^{-}\right\|=\sqrt{\Delta {\tilde{x}}_{k_{1}}^{2}+\Delta {\tilde{x}}_{k_{2}}^{2}+\Delta {\tilde{x}}_{k_{3}}^{2}+\ldots +\Delta {\tilde{x}}_{k_{m}}^{2}}$$

where m is the total number of selected state components, and $\Delta {\tilde{x}}_{k_{m}}^{2}$ is the discrepancy values of state components. A smaller $\alpha_{k}$ amplifies the predicted covariance and increases the Kalman gain, which raises the weight of GNSS observations and reduces confidence in INS dynamic prediction. Threshold widening reduces the sensitivity of $\alpha_{k}$ to state discrepancy, making $\alpha_{k}$ more likely to remain close to 1. $c_{0}^{*}\left(k\right)$ and $c_{1}^{*}\left(k\right)$ are the dynamic adaptive thresholds, which are generated by the following expressions:

(36) $$\left\{\begin{array}{c} c_{0}^{*}\left(k\right)=c_{0,base}\cdot \beta \left(k\right)\\ c_{1}^{*}\left(k\right)=c_{1,base}\cdot \beta \left(k\right) \end{array}\right.$$

where $c_{0,base}$ and $c_{1,base}$ are two constant thresholds, typically chosen within the ranges $c_{0,base}=1.0∼1.5$ and $c_{1,base}=3.0∼8.5$; $\beta \left(k\right)$ is defined as a comprehensive adjustment factor, constructed as follows:

(37) $$\beta \left(k\right)=1+\eta_{d}\cdot I_{d}\left(k\right)+\eta_{g}\cdot I_{g}\left(k\right)$$

where $\eta_{d}$ and $\eta_{g}$ are non-negative weighting coefficients that determine the extent to which vehicle dynamics and observation quality affect threshold relaxation. They are set to 0.8 and 0.6, respectively, in this study, and can be adjusted for specific application scenarios. The dynamic intensity $I_{d}\left(k\right)$ and the geometric precision factor $I_{g}\left(k\right)$ are defined as follows:

(38) $$\left\{\begin{array}{c} I_{d}\left(k\right)=\min\left[\begin{array}{cc} \frac{\sigma_{a}^{2}\left(k\right)}{T_{d}}, & 1 \end{array}\right]\\ I_{g}\left(k\right)=\min\left[\begin{array}{cc} \frac{PDOP\left(k\right)}{PDOP_{g}}, & 1 \end{array}\right] \end{array}\right.$$

where $T_{d}$ is an empirical threshold for classifying dynamics. A value of 0.94 m^2^/s^4^ is adopted in this work when using the variance of three-dimensional accelerometer measurements. A value of 0.34 m^2^/s^4^ applies for dynamic detection based on horizontal accelerometer data. $PDOP_{g}$ is the PDOP threshold for good conditions. A reasonable range of 2.0 to 3.0 is generally recommended for urban dynamic positioning scenarios, and a value of 2.5 is used in this study. In general, threshold relaxation increases the relative influence of INS prediction and reduces the weight of GNSS observations in the state update. $\sigma_{a}^{2}\left(k\right)$ denotes the variance within a sliding time window. The window length is set to 50 epochs in this study, which equals a time span of 1 s at the IMU sampling rate of 50 Hz. Its calculation formula is given by:

(39) $$\sigma_{a}^{2}\left(k\right)=\tau \cdot \sigma_{a}^{2}\left(k-1\right)+\left(1-\tau \right)\cdot Var\left[a_{b}^{\left(k\right)}\right]$$

where τ is the first-order low-pass filter coefficient, and $a_{b}^{\left(k\right)}$ denotes the accelerometer measurement vector expressed in the b-frame. When the vehicle is in a highly dynamic state, the variance of accelerometer measurements increases significantly. The dynamic intensity term approaches 1, and the comprehensive adjustment factor rises accordingly. The widened adaptive threshold prevents normal state deviations caused by vehicle maneuvers from being misclassified as model mismatch. When satellite geometry is poor, PDOP increases, and the geometric precision factor term approaches 1. The elevated threshold reduces the sensitivity of the adaptive factor to the state deviation statistic. For scenarios where PDOP is unavailable, the geometric precision factor is set to 1 by default. Figure 2a shows the three-axis accelerometer measurements of Xiaomi Mi 8, while Figure 2b presents the corresponding variance results with the filter coefficient τ set to 0.95. While the vehicle is stationary or moving at a constant speed, the variance remains at a relatively low level. When the vehicle changes direction rapidly, the variance curve rises rapidly. By comparing this variance with the empirical threshold, the dynamic level of the vehicle in the current epoch can be classified.

**Figure 3:**
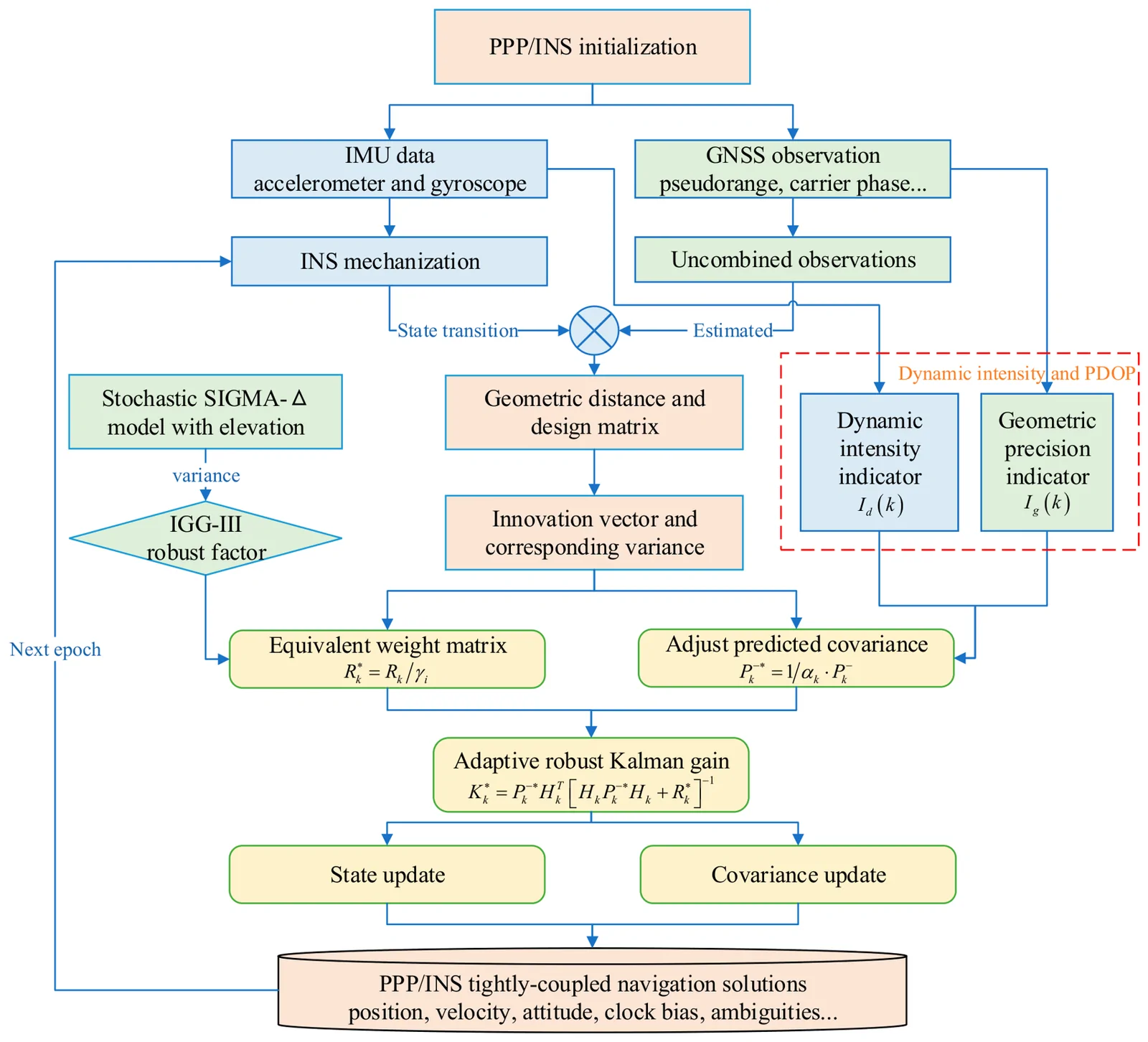
Flow diagram of the proposed tightly coupled PPP/INS integration with an adaptive robust Kalman filter based on vehicle dynamic intensity and PDOP.

Figure 3 shows the workflow of the proposed tightly coupled PPP/INS integration based on an adaptive robust Kalman filter. The time update method of standard EKF is adopted in the prediction stage, and the prior state and covariance are provided by the INS mechanization. In the update stage, the tightly coupled architecture is adopted, where INS predicted states are mapped to the predicted pseudorange and phase values, and the innovation vector is constructed as the difference between raw GNSS observations and these predicted values. The IGG-III weighting function is applied to down-weight abnormal GNSS observations, and an equivalent weight matrix is constructed to suppress the effects of multipath and NLOS. A comprehensive adjustment factor based on the dynamic intensity and PDOP is constructed to enable dynamically adjust of the adaptive threshold.

## 4. Experiment and Results

### 4.1. Smartphone GNSS and IMU Data Collection

**Figure 4:**
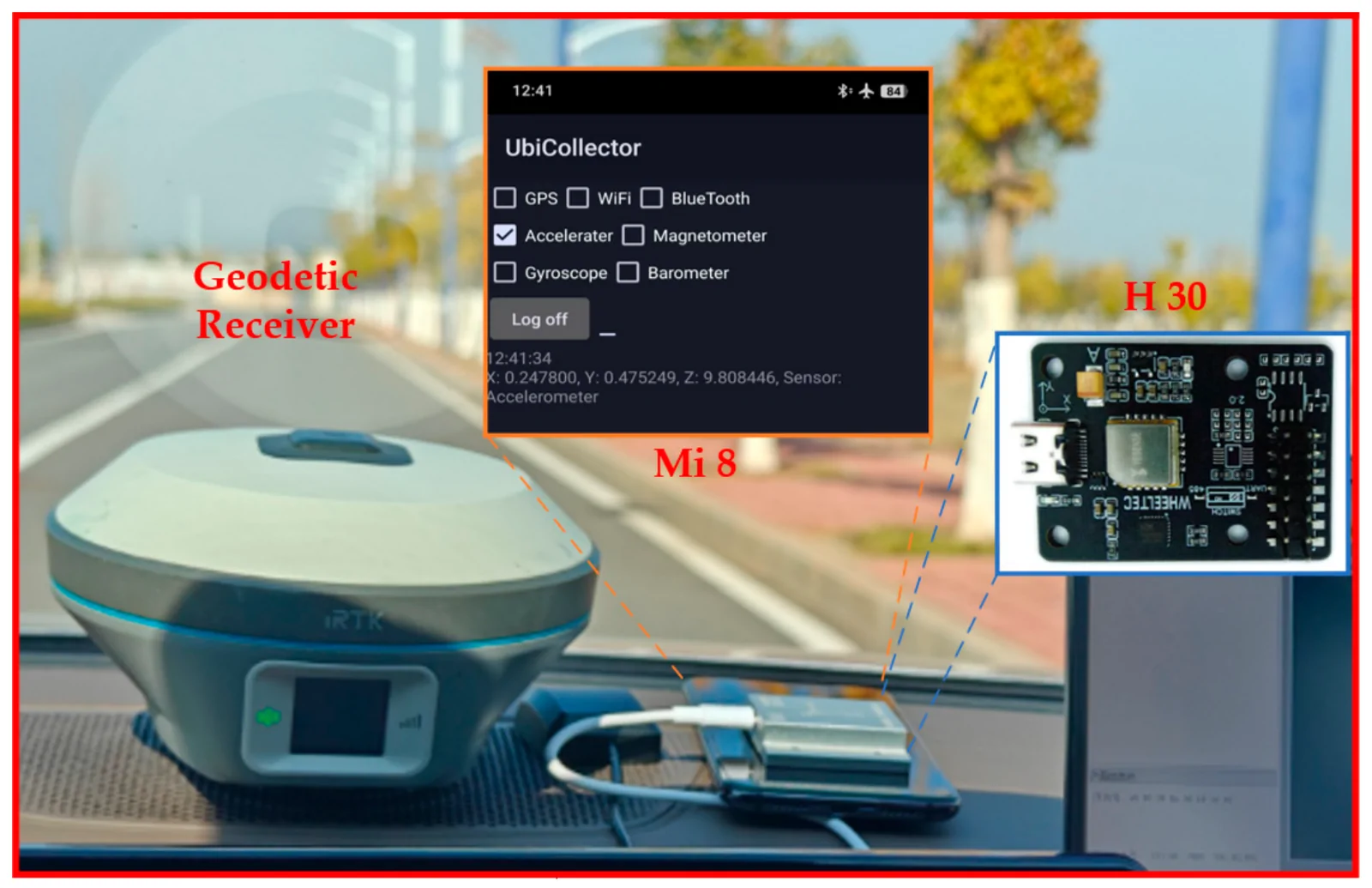
View of the experimental devices. The application interface of the integrated smartphone sensor data collection is shown in orange box. The uncased external IMU model H30 is shown in blue box.

To evaluate the performance of the proposed tightly coupled PPP/INS adaptive robust Kalman filter algorithm for smartphones, two comparative experiments were conducted in vehicle kinematic scenarios. In these experiments, smartphones were placed on the vehicle’s center console, which represents a typical navigation application scenario, as shown in Figure 4. By comparing the positioning results with those from a high-precision geodetic receiver, we can quantitatively analyze the positioning accuracy, stability and availability of the proposed algorithm.

Figure 4 shows a geodetic-grade GNSS receiver on the left, which provides reference positions for comparison via carrier phase post-processing kinematic (PPK) positioning. The Xiaomi Mi 8 is adhered and fixed at the center, integrated Broadcom BCM47755 GNSS chipset, which supports dual-frequency signal reception, and integrates the ICM-20690 MEMS IMU. The initial zero rate output (ZRO) bias of the three-axis gyroscope of ICM-20690 is ±1 deg/s, the rate noise density is 0.004 deg/s/√Hz, while the initial bias of the three-axis accelerometer is ±40 mg and the noise density is 0.1 mg/√Hz. Meanwhile, an external consumer-grade IMU WHEELTEC H30 is added for comparison. The bias stability of the three-axis gyroscope of H30 is ±4 deg/h, and the rate noise density is 0.005 deg/s/√Hz, while the bias stability of the accelerometer is ±0.035 mg and the noise density is 60 µg/√Hz. The GNSS observations are collected using the application named GEO++ RINEX Logger 2.1.6, while the IMU data are obtained through the self-developed program. It should be noted that the solutions for all the following experiments are based on the stochastic SIGMA-Δ model. For epochs with unavailable PPK solutions, the reference coordinates were obtained directly via interpolation.

### 4.2. PPP/INS Solutions on the Urban Expressway

**Figure 5:**
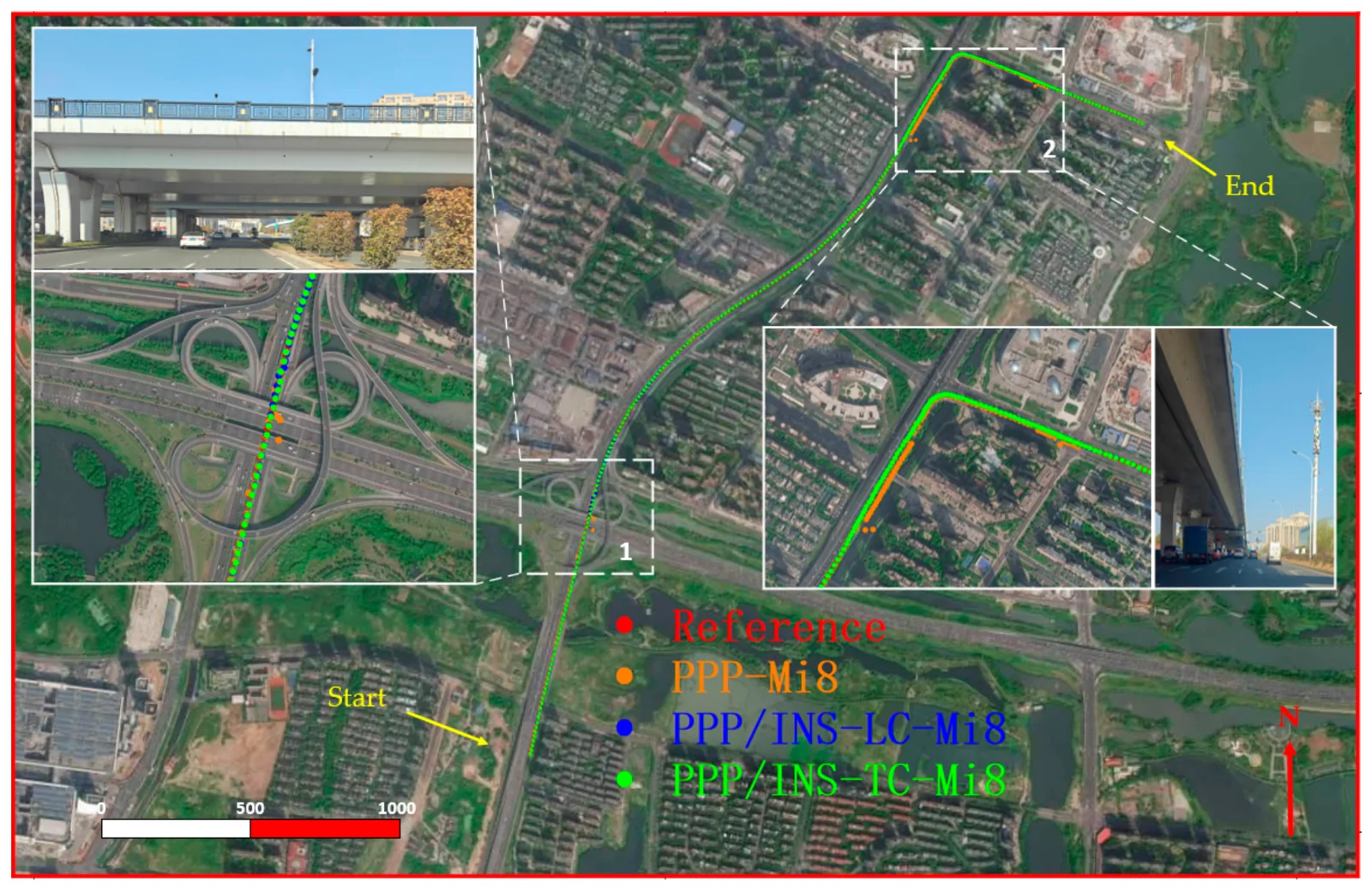
Trajectories of Xiaomi Mi 8 using PPP, loosely coupled PPP/INS, and the proposed tightly coupled PPP/INS solutions on the urban expressway. The white boxes 1 and 2 indicate the corresponding real-world environments.

The first experiment was conducted on an urban expressway section in Nanchang, Jiangxi Province, China. The total trajectory length was approximately 3.4 km, as shown in Figure 5. The route started on a ground expressway, passed through an overpass to enter an elevated road section marked by white box 1. Subsequently, the vehicle exited via the right-side ramp marked by white box 2, and finally turned right to reach the endpoint. This experiment used dual-frequency GPS/BDS/GLONASS observations from the Xiaomi Mi 8 and measurements from its built-in IMU chipset ICM-20690. The positioning solutions included the uncombined PPP method, the loosely coupled PPP/INS integration, and the proposed adaptive robust tightly coupled PPP/INS integration. The INS was initialized with a velocity of 5 m/s.

**Figure 6:**
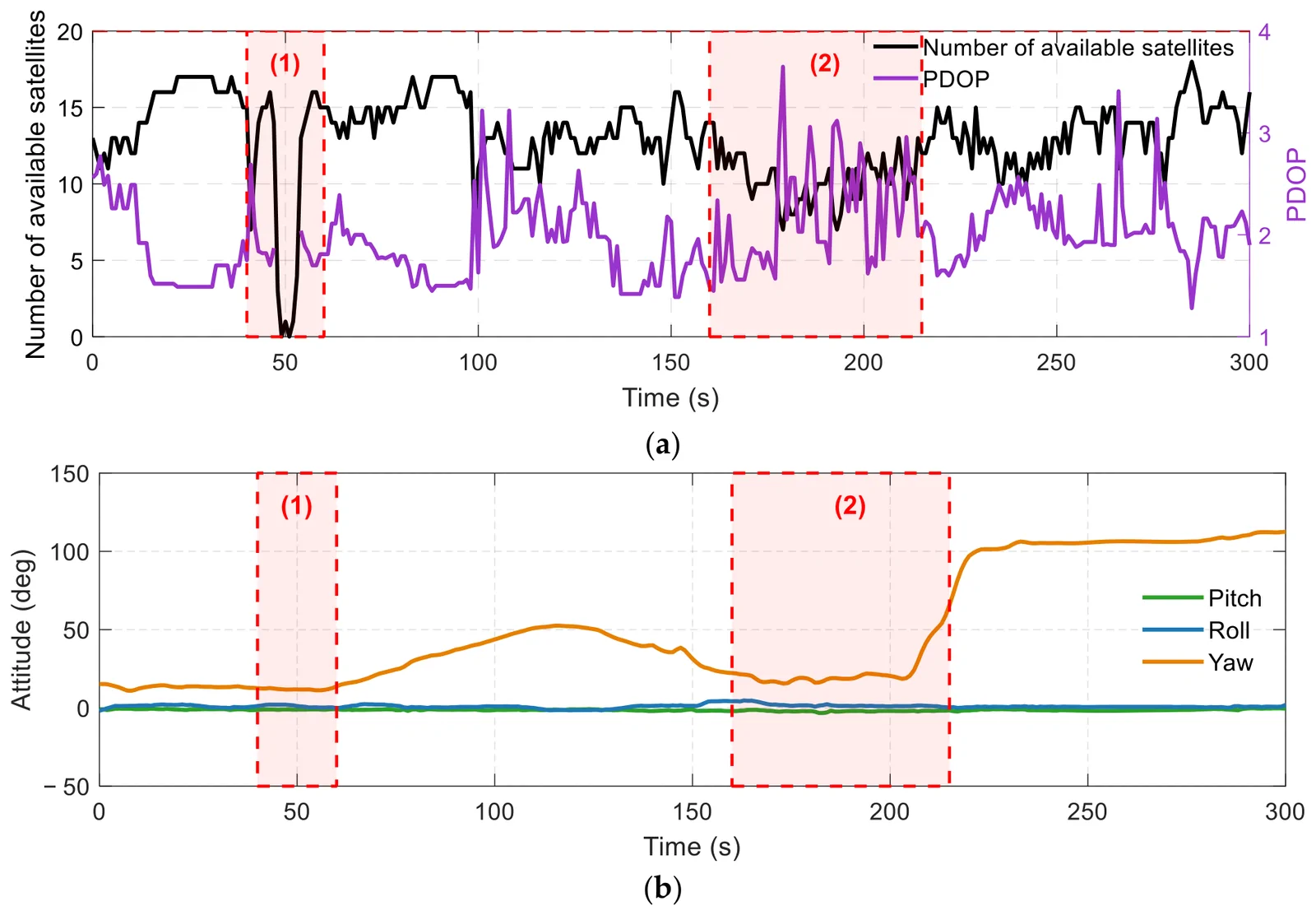
Number of available satellites, PDOP values, and the three attitude angles of Xiaomi Mi 8 on the urban expressway. (**a**) Available satellites and PDOP; (**b**) attitude angles (pitch, roll, yaw). The red boxes 1 and 2 correspond to white boxes 1 and 2 in Figure 5, respectively.

Among the three solutions, the PPP solution exhibits significant gaps and deviations from the reference trajectory in open environments such as urban elevated expressways. In contrast, both the loosely coupled and tightly coupled PPP/INS solutions align well with the reference trajectory. The IMU-aided PPP method has advantages in terms of navigation accuracy and continuity in areas where signals are blocked. Figure 6 shows the number of available satellites, PDOP values, and variations in the three attitude angles during the experiment. 

**Figure 7:**
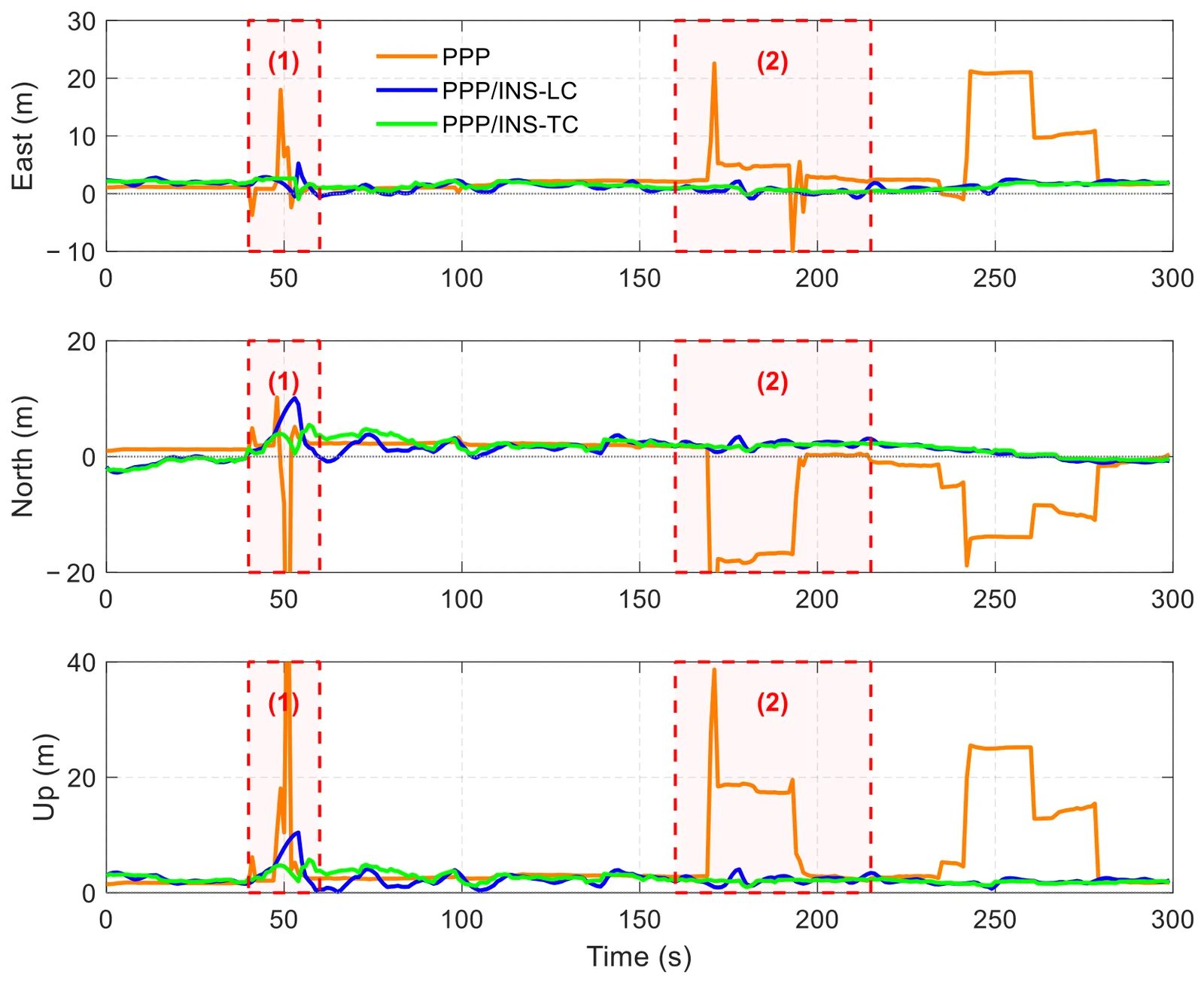
Positioning errors of the Xiaomi Mi 8 PPP, loosely coupled PPP/INS, and the proposed tightly coupled PPP/INS solutions on the urban expressway. The red boxes 1 and 2 correspond to white boxes 1 and 2 in Figure 5, respectively.

As shown in Figure 6, satellite visibility and PDOP values are affected by surrounding tall structures, overpasses and elevated roads, even on urban expressways. When passing under the overpass marked by red box 1, the number of available satellites decreased from 14 at epoch 48 to 3 at epoch 49, and further dropped to 0 at epoch 50. Subsequently, only 1 satellite was intermittently observed; 4 satellites were available at epoch 54, and the number recovered to 13 at epoch 55. After leaving the elevated expressway marked by the red box 2, the number of available satellites decreased significantly again due to obstruction caused by the elevated road on the left. The minimum number of satellites was observed at epoch 180, with 7 satellites available and a PDOP value of 3.65. Furthermore, the variations in pitch and roll angles are relatively smooth, and the yaw angles are generally consistent with the heading direction of the vehicle. With post-processed PPK coordinates taken as the reference, the positioning errors of Xiaomi Mi 8 PPP, loosely coupled PPP/INS, and the proposed tightly coupled PPP/INS solutions in the east, north, and horizontal directions are shown in Figure 7. 

**Figure 8:**
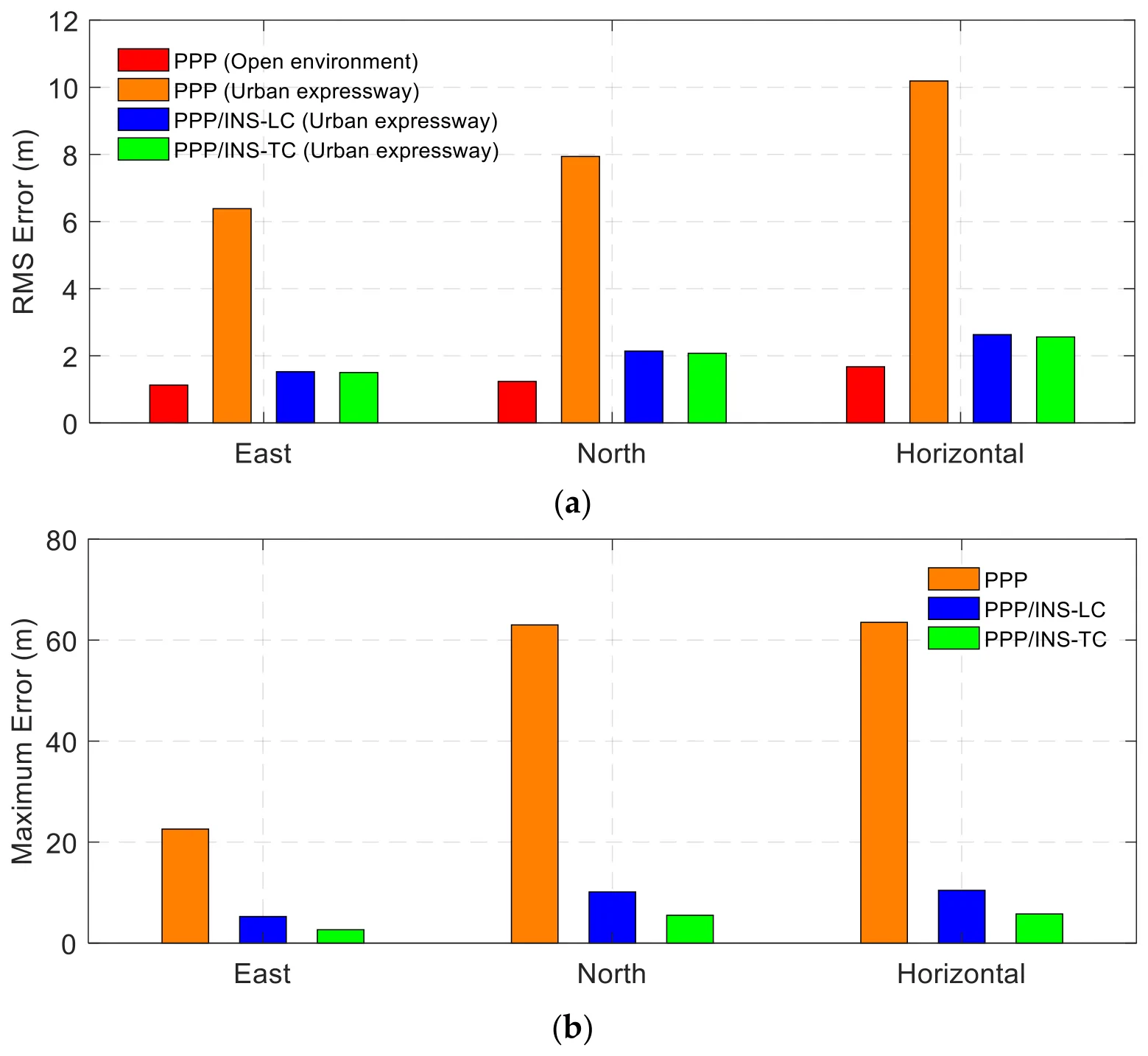
RMS and maximum errors of PPP, loosely coupled PPP/INS, and the proposed tightly coupled PPP/INS solutions for the Xiaomi Mi 8 on the urban expressway. (**a**) RMS error; (**b**) maximum error.

During the first 40 epochs of the experiment, the vehicle traveled in an open environment with no significant obstructions. The positioning RMS of the PPP solution in this open segment was compared with the results of all solutions over the entire expressway section, as shown in Figure 8a. In the area marked by red box 1 in Figure 7, the PPP solution suffered severe performance degradation due to insufficient observations, its east direction positioning error increased rapidly, with a maximum value exceeding 18 m. For the loosely coupled PPP/INS solution, positioning outputs are recursively estimated by the INS when most GNSS signals are blocked. The maximum east direction error was reduced to 5.262 m, as shown in Figure 8b. However, the PPP/INS-LC positioning error accumulates rapidly when the number of satellites is insufficient to support standalone PPP. In contrast, the proposed tightly coupled PPP/INS solution can perform coupled positioning with only a small number of GNSS observations; even the signals are severely blocked, effectively suppressing the drift of positioning errors. Its maximum east direction error was reduced to 2.653 m.

In the area marked by the red box 2, the positioning accuracy of the PPP solution decreased significantly. The maximum horizontal error exceeded 38 m, with an overall offset of approximately 5 m in the east direction and 17 m in the south direction. This systematic deviation may be caused by satellite signal blockage from the elevated road on the northwest side. In contrast, the positioning errors of the loosely coupled PPP/INS and the proposed tightly coupled PPP/INS solutions were significantly reduced. The tightly coupled PPP/INS solution exhibited stronger robustness, with a maximum horizontal error of 2.279 m, which is lower than the 4.065 m of the PPP/INS-LC scheme.

As shown in Table 1, during the first 40 epochs of the experiment, the horizontal positioning RMS of the Xiaomi Mi 8 PPP solution is 1.675 m. This result demonstrates that the dual-frequency multi-GNSS observations from smartphones can deliver approximately 1.5 m level kinematic positioning in open urban environments. However, the positioning performance of the PPP solution deteriorates significantly in areas where the signal is obstructed, with a horizontal RMS error exceeding 10 m. Compared with PPP solution, the accuracy of the loosely coupled PPP/INS scheme is significantly improved, with the horizontal RMS error reduced by 74.17% to 2.632 m. Meanwhile, the positioning performance of the proposed tightly coupled PPP/INS is further enhanced, with a horizontal RMS error of 2.563 m. The main advantage of the tightly coupled architecture in this test lies in the suppression of extreme positioning errors and improving positioning continuity under short-term signal obstruction. The maximum error of the PPP solution reaches tens of meters, indicating that severe signal blockage or loss of phase lock can cause extreme positioning divergence. The loosely coupled PPP/INS reduces the maximum error to 10.449 m, while the proposed tightly coupled PPP/INS further decreases the maximum error by 44.82% to 5.766 m. It should be noted that the three schemes compared in this experiment are standalone PPP, conventional loosely coupled PPP/INS, and the proposed adaptive robust tightly coupled PPP/INS. The performance improvement is mainly attributed to INS aiding and the tight coupling architecture. In the following section, the tightly coupled PPP/INS with the adaptive robust Kalman filter is tested in complex urban road environments. The solutions is further divided into two variants, robust tightly coupled PPP/INS and adaptive robust tightly coupled PPP/INS, to quantify their respective impacts on positioning results.

### 4.3. PPP/INS Solutions for Urban Complex Roads and Tunnel

**Figure 9:**
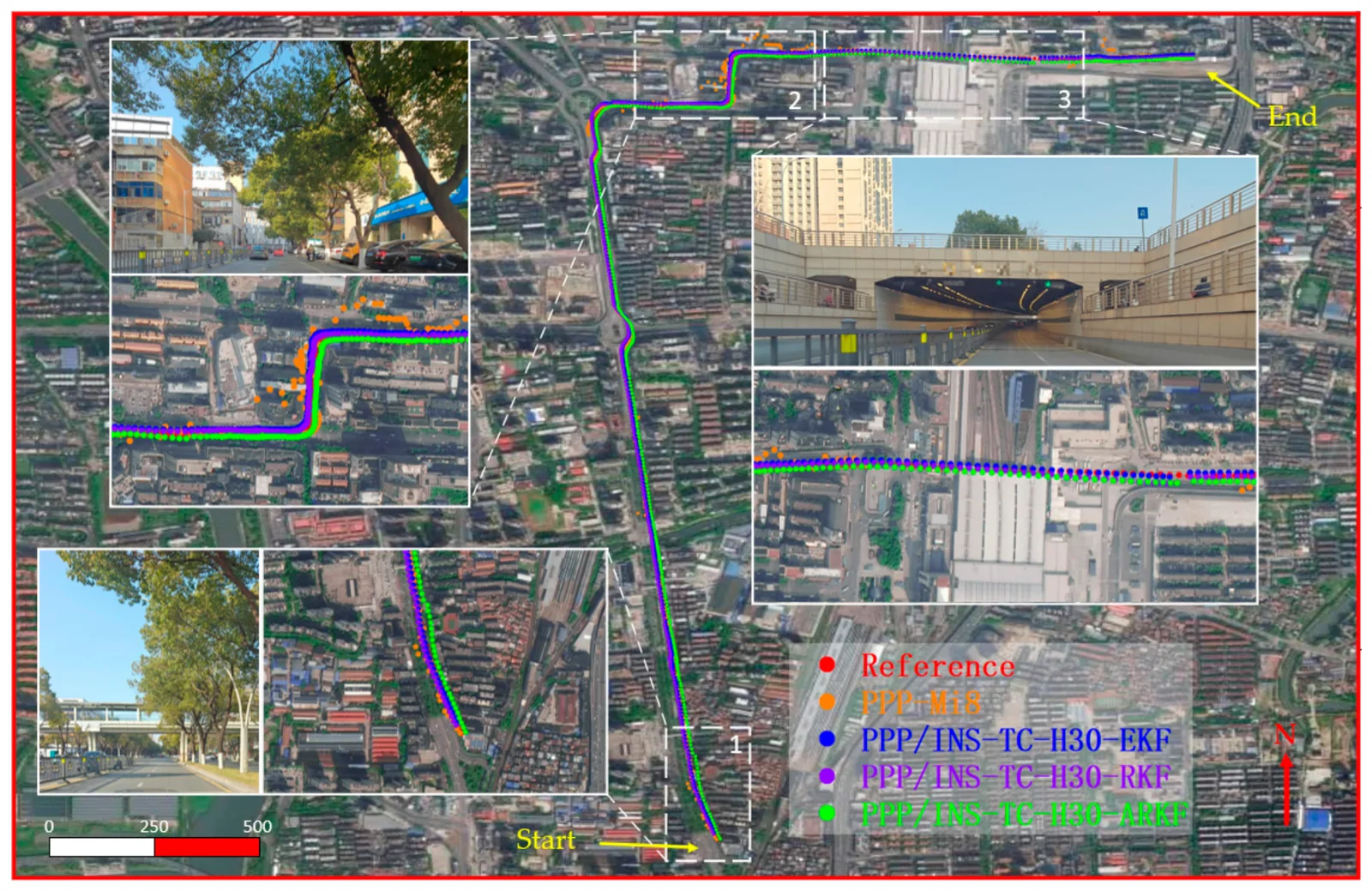
Trajectories of the four solutions using Xiaomi Mi 8 and the H30 IMU in complex urban roads and a tunnel. The white box 1, 2, and 3 indicate the corresponding real-world environments.

The second experiment was conducted on the main and branch roads of the city, with a total trajectory length of approximately 4.5 km. As shown in Figure 9, the route passes through various complex environments, including a tree-lined road, an urban branch road, and a long tunnel. Three typical regions are highlighted in white boxes: the tree-lined section marked by box 1, the urban branch road marked by box 2, and the tunnel section marked by box 3. This experiment adopted the tightly coupled PPP/INS integration scheme, combining an external consumer-grade IMU H30 with the Xiaomi Mi 8 GNSS chipset. The EKF-based tightly coupled PPP/INS used the conventional filtering configuration; the robust Kalman filter (RKF)-based tightly coupled PPP/INS introduced a robust factor calculated from normalized innovations via the IGG-III weight function to suppress outliers; the ARKF-based tightly coupled PPP/INS integrated the proposed adaptive factor, which is driven by dynamic intensity and geometric precision indicators.

**Figure 10:**
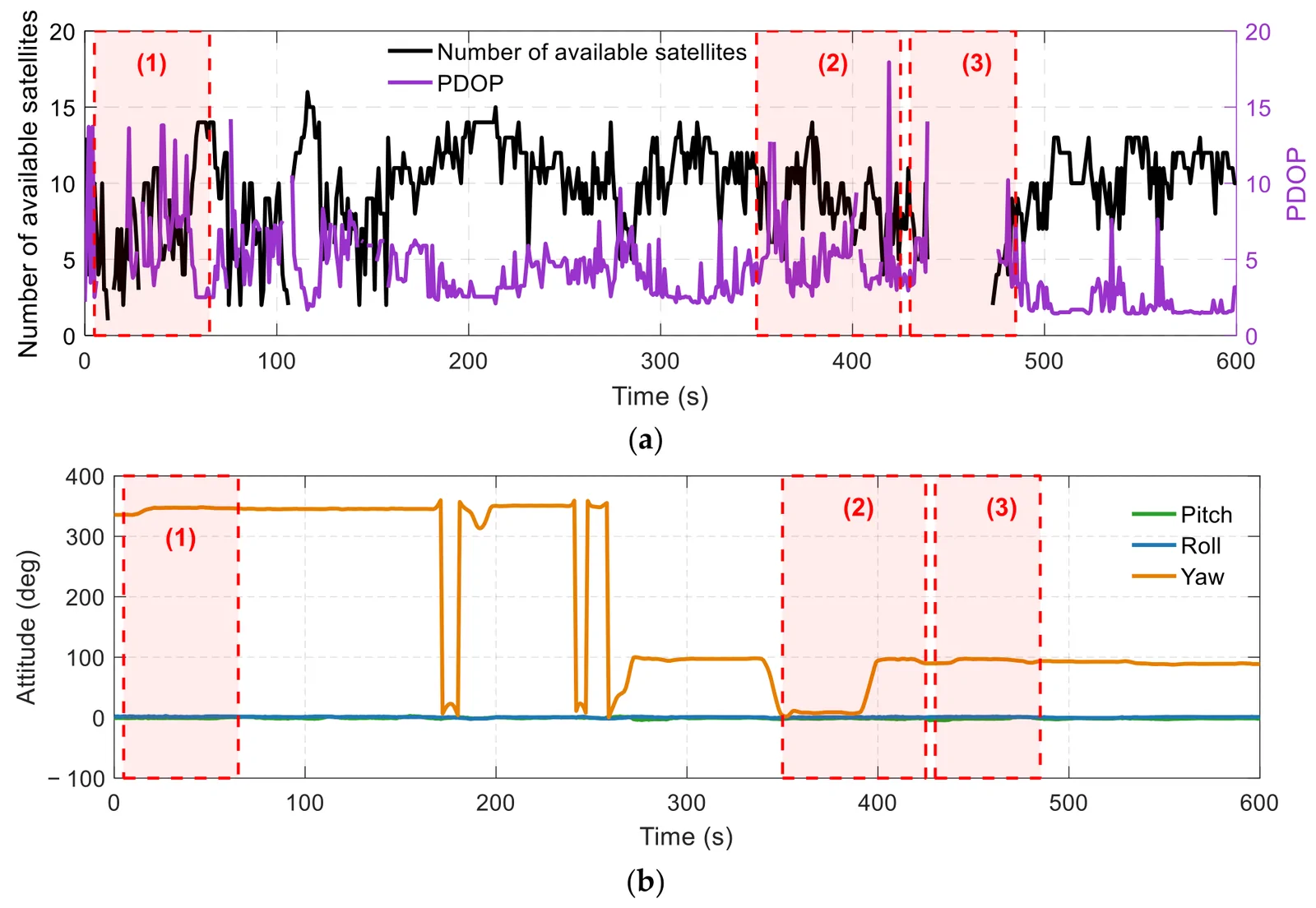
Number of available satellites, PDOP values, and three attitude angles in complex urban roads and a tunnel. (**a**) Available satellites and PDOP; (**b**) attitude angles (pitch, roll, yaw). The red boxes 1, 2, and 3 correspond to white boxes 1, 2, and 3 in Figure 9, respectively.

Compared with the urban expressway experiment, positioning trajectory fluctuations are significantly larger in complex urban environments. In particular, the PPP solution showed large deviations in the urban branch road marked by box 2. As shown in Figure 10, fewer than 10 satellites were available for more than half of the experimental epochs. On the tree-lined road and urban branch road, the number of available satellites sometimes dropped to fewer than 4, or even resulted in complete loss of signal lock. In tunnel sections, GNSS signals were fully blocked and PDOP values became unavailable. The yaw angle varied from 0 to 360 degrees and was generally consistent with the actual motion of the vehicle.

**Figure 11:**
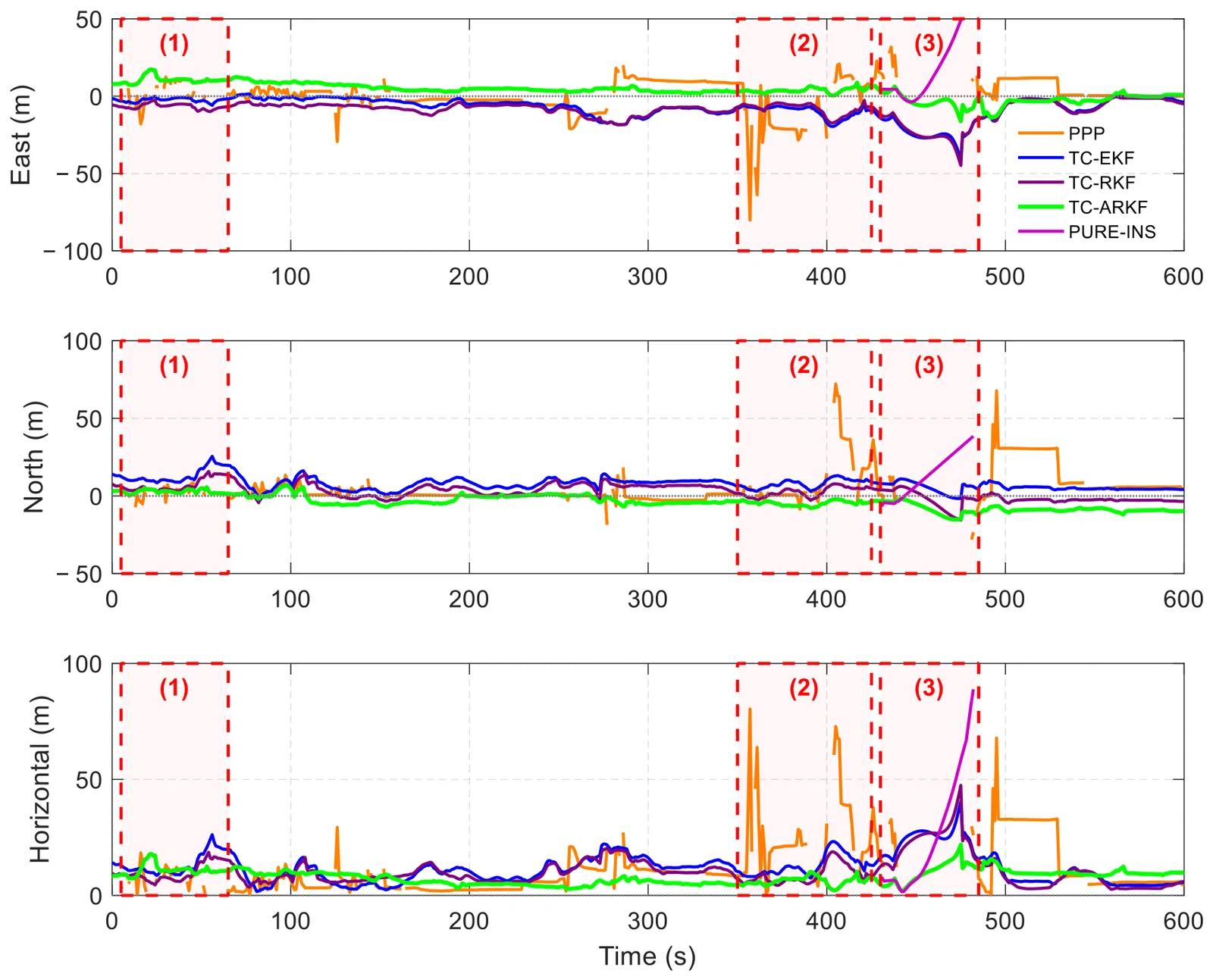
Positioning errors of PPP, TC-EKF, TC-RKF, TC-ARKF and pure INS solutions for the Xiaomi Mi 8 in complex urban roads and a tunnel. The red boxes 1, 2, and 3 correspond to white boxes 1, 2, and 3 in Figure 9, respectively.

The GNSS signals are frequently blocked by trees and surrounding buildings in complex urban environments, and the number of available satellites may sometimes fail to meet the requirements for PPP. Excluding the tunnel section, there were still 49 epochs during which PPP solutions could not be generated, as shown in Figure 11. In contrast, the EKF-based tightly coupled PPP/INS can provide continuous positioning outputs. However, since the built-in omnidirectional linearly polarized antenna of smartphones is susceptible to multipath and other interference, the horizontal positioning error of TC-EKF still exceeds 25 m. The RKF-based tightly coupled PPP/INS reduces the impact of abnormal observations by using a robust factor to dynamically adjust the weights of the observations affected by multipath. Nevertheless, the TC-RKF scheme still has limited on improving positioning stability, with a maximum error of over 20 m in complex urban road scenarios. The proposed ARKF-based tightly coupled PPP/INS adjusts the filter confidence coefficient via an adaptive factor constructed from dynamic intensity and geometric precision indicators, optimizes the Kalman gain, and significantly improves the positioning stability.

The length of the tunnel is approximately 500 m, and the GNSS signal outage lasts for about 41 epochs. Under complete signal outage conditions, pure inertial navigation suffers from rapid error accumulation due to the drift of low-cost IMU sensors. As shown by the PURE-INS curve in Figure 11, the horizontal positioning error diverges significantly inside the tunnel, reaching a maximum value of 88.834 m across the entire tunnel section. All three tightly coupled PPP/INS schemes achieve notably higher accuracy than pure INS. However, compared with TC-EKF and TC-RKF, the proposed TC-ARKF provides more accurate position and velocity estimates at the tunnel entrance, which is beneficial for reducing the accumulation of errors.

**Figure 12:**
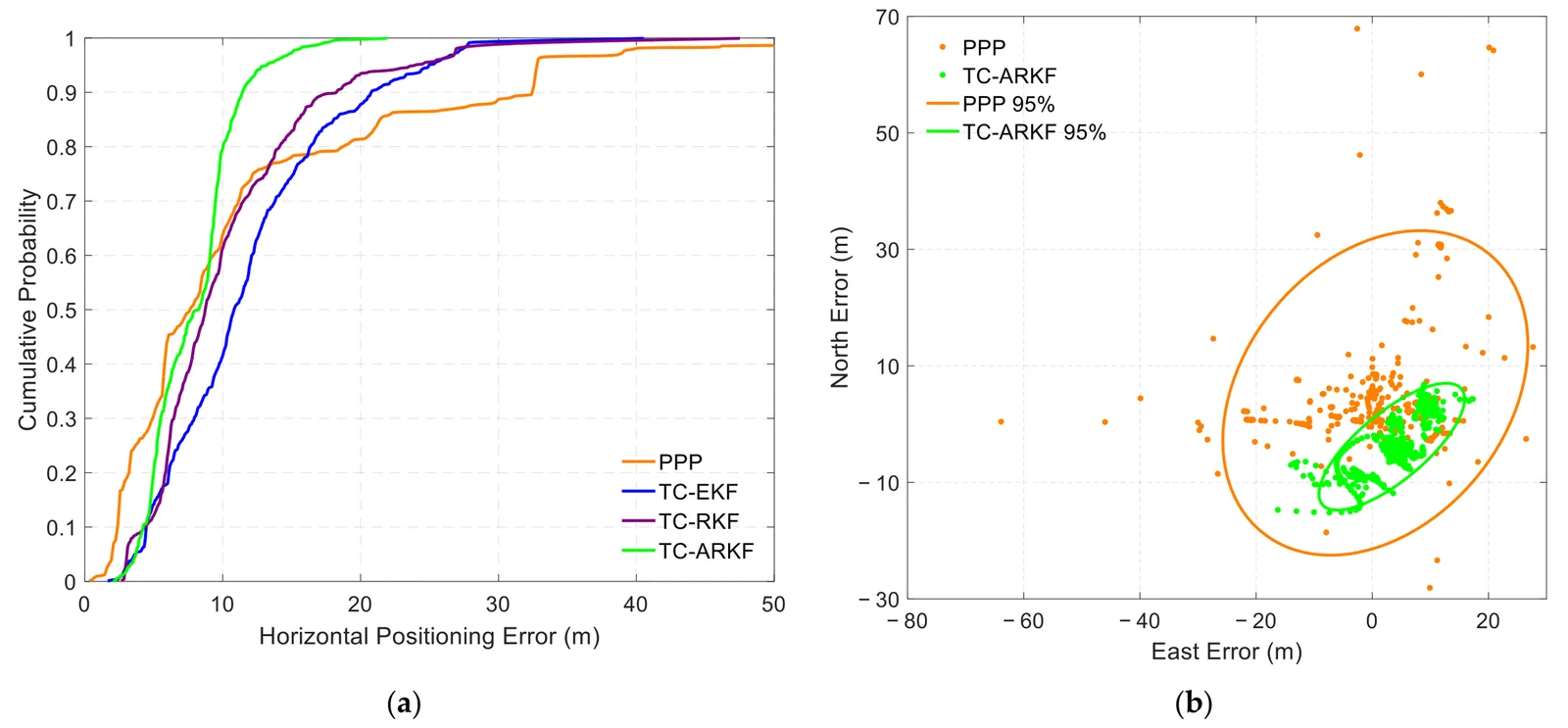
CDF curves and ellipse of horizontal positioning errors of the Xiaomi Mi 8 in complex urban roads and a tunnel. (**a**) CDF of horizontal positioning errors; (**b**) 95% horizontal error ellipse.

Figure 12 shows the cumulative distribution function (CDF) of horizontal positioning errors for four solutions and the 95% confidence error ellipses for PPP and TC-ARKF. Although the PPP CDF curve appears further to the left, this is because it only covers epochs for which valid PPP solutions could be generated. For the error ellipse, the area of the 95% error ellipse for the TC-ARKF solution is only 12.94% of that for the PPP solution. Meanwhile, among the three PPP/INS solutions, the CDF curve of the proposed TC-ARKF lies furthest to the left at both the 68% and 95% cumulative probability levels, demonstrating that this method achieves the best positioning accuracy and stability in complex urban environments.

As shown in Table 2, the horizontal positioning RMS of TC-ARKF is 8.492 m in complex urban road and tunnel environments, significantly outperforming the TC-EKF with the RMS of 13.434 m and the TC-RKF with the RMS of 12.042 m. The comparison between TC-EKF and TC-RKF demonstrates the contribution of the robust estimation module. The robust equivalent weight function downweights observations affected by multipath and NLOS errors. This reduces the horizontal RMS error from 13.434 m to 12.042 m, corresponding to an accuracy improvement of 10.36%. It should be noted that the maximum horizontal error of TC-RKF in the tunnel section reaches 47.504 m, which is slightly larger than 40.540 m of TC-EKF. This occurs because robust estimation may downweight partially valid observations during the gradual signal fading stage at the tunnel entrance. The comparison between TC-RKF and TC-ARKF reflects the contribution of the proposed adaptive factor adjustment method. The dynamically adjusted adaptive factor optimizes the weight allocation between the predicted state and observations according to vehicle dynamic intensity and satellite geometry. This further reduces the horizontal RMS error from 12.042 m to 8.492 m, with an additional accuracy improvement of 29.48%. In tunnel area where the GNSS signals are blocked, the horizontal positioning RMS of pure INS reaches 39.653 m, and its maximum horizontal error reaches 88.834 m throughout the tunnel. All three tightly coupled PPP/INS solutions maintain significantly higher positioning accuracy than pure INS. This advantage is rooted in accurate navigation states and IMU bias estimates established by GNSS observations at the tunnel entrance, which constrained inertial error propagation throughout the signal outage. Among the three tightly coupled solutions, the maximum horizontal error of TC-ARKF is 21.918 m, which is significantly lower than that 40.540 m of TC-EKF and 47.504 m of TC-RKF, corresponding to reductions of approximately 45.93% and 53.86%, respectively.

## 5. Conclusions

This paper proposes a tightly coupled PPP/INS integration method based on an adaptive robust Kalman filter to address the problems of GNSS signal occlusion and severe multipath effects for smartphone positioning in complex urban environments. By integrating raw GNSS observations with IMU measurements of smartphones within an extended Kalman filter framework, the proposed method integrates a robust estimation factor based on the IGG-III weight function and a new adaptive factor derived by dynamic intensity and geometric precision indicators, effectively suppressing observation outliers and dynamic model errors. To validate the performance of the proposed algorithm, two vehicle dynamic experiments were conducted using a Xiaomi Mi 8 smartphone and an external H30 IMU. One was tested on an urban expressway, and the other was tested in complex urban road and tunnel scenarios. The main conclusions drawn from the experiments are as follows:

(1) In the urban expressway experiment, smartphone PPP achieves a horizontal positioning RMS of 1.675 m in the open-sky environments of the first 40 epochs. This result indicates that dual-frequency multi-GNSS observations from smartphones can deliver approximately 1.5 m level kinematic positioning under favorable open urban conditions. The introduction of INS coupling effectively reduces the large fluctuations in PPP errors. Specifically, the loosely coupled PPP/INS solution reduces the horizontal RMS from 10.188 m to 2.632 m, corresponding to an improvement of 74.17%. The tightly coupled PPP/INS solution further reduces the horizontal RMS to 2.563 m, with limited improvement in overall accuracy compared with the loosely coupled scheme. In terms of extreme error suppression, the tightly coupled PPP/INS solution reduces the maximum positioning error from 10.449 m for the loosely coupled scheme to 5.766 m, a reduction of 44.82% in maximum error. These results show that the tightly coupled PPP/INS model outperforms the loosely coupled solution in terms of positioning continuity and robustness against short-term signal blockage under the tested urban expressway scenarios with intermittent obstructions.
(2) In the complex urban road experiment, extreme environments such as frequent GNSS signal blockage and severe multipath effect posed significant challenges to the navigation system. The horizontal RMS of smartphone PPP exceeds 15 m. The tightly coupled PPP/INS model based on RKF parameter estimation reduces the errors caused by multipath effects, achieving a horizontal RMS of 12.042 m. In contrast, the proposed tightly coupled PPP/INS solution with ARKF further optimized the Kalman gain based on geometric precision and dynamic intensity indicators, achieving a horizontal RMS of 8.492 m, which represents a 36.79% improvement over the conventional EKF-based tightly coupled scheme.
(3) The tunnel experiment demonstrates that the tightly coupled PPP/INS solution based on ARKF achieves a maximum horizontal positioning error of 21.918 m throughout the approximately 500 m tunnel section. Benefiting from a superior state parameter established before entering the tunnel, the method effectively reduced the drift of INS errors. In complex environments, the constructed adaptive factor adjusts the weight allocation of the filter between GNSS observations and the INS dynamic model according to real-time dynamic intensity and geometric precision indicators. Therefore, the proposed method maintains better positioning continuity and stability in urban environments such as GNSS signal degradation and short-term outages.

The experimental results show that the proposed ARKF-based tightly coupled PPP/INS scheme achieves better overall performance than standard EKF-based and RKF-based tightly coupled schemes under the tested urban scenarios. It presents notable advantages in maximum error suppression and positioning stability. The experiments also reveal that the tightly coupled PPP/INS integration model is more susceptible to satellite signal quality during the initial stage, and that INS drift remains significant in long tunnel sections. The proposed adaptive robust Kalman filter operates recursively and is structurally suitable for real-time applications. However, performance discrepancies across different Android smartphones must be taken into account in positioning computation. Specifically, older-generation smartphone models only support single-frequency GNSS measurements, and performance variations in built-in inertial measurement units (IMUs) lead to non-uniform output sampling rates. Furthermore, the activation of duty-cycle mode also has a direct impact on positioning accuracy. Future work will continue to develop a real-time integrated navigation application for smartphone platforms, investigate a deep learning-based adaptive factor tuning model, and integrate additional sensors such as visual sensors to construct a robust positioning framework applicable to a wider range of urban environments. Moreover, with the continuous advancement of GNSS and IMU technologies, high-precision positioning for low-cost consumer devices is expected to play an increasingly critical role in applications including intelligent transportation systems (ITS), location-based services (LBS), and autonomous driving.

## Figures and Tables

**Figure 2:**
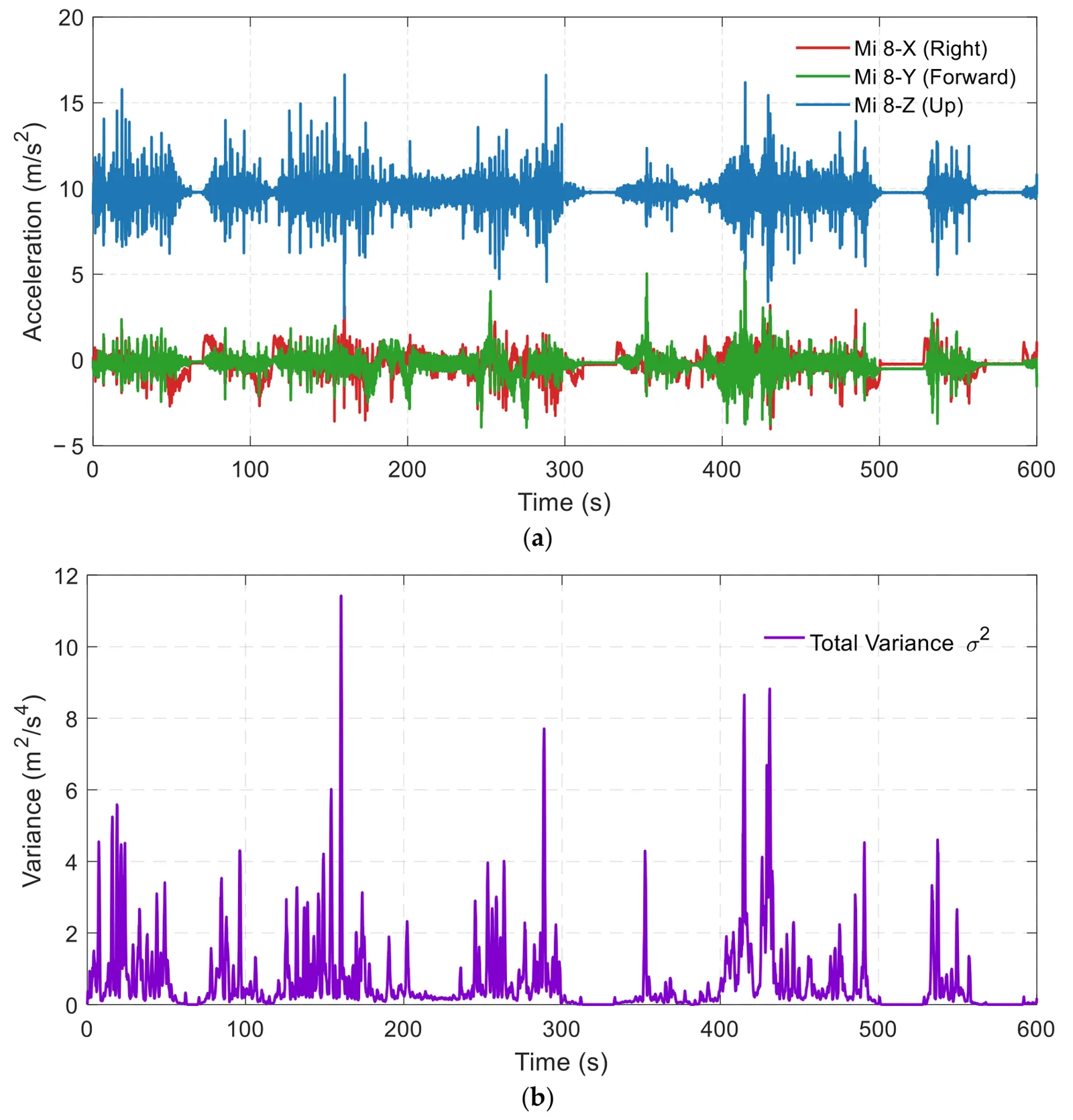
The measurements and variance of the three-axis accelerometer of Xiaomi Mi 8. (**a**) Three-axis accelerometer measurements; (**b**) Corresponding variance of accelerometer measurements.

**Table 1:** RMS of PPP, loosely coupled PPP/INS, and the proposed tightly coupled PPP/INS positioning errors for the Xiaomi Mi 8 on the urban expressway. H denotes the horizontal direction.

Direction	Mi 8 GPS/BDS/GLONASS Kinematic Positioning Error RMS (m)			
	PPP (1–40 Epochs)	PPP	PPP/INS-LC	PPP/INS-TC
E	1.128	6.384	1.528	1.502
N	1.238	7.940	2.143	2.076
H	1.675	10.188	2.632	2.563
Max (H)	1.781	63.527	10.449	5.766

**Table 2:** RMS of positioning errors for the PPP, TC-EKF, TC-RKF, and TC-ARKF schemes using the Xiaomi Mi 8 in complex urban roads and a tunnel.

Direction	Mi 8 GPS/BDS/GLONASS Kinematic Positioning Error RMS (m)				
	PPP	PPP/INS-TC-H30-EKF	PPP/INS-TC-H30-RKF	PPP/INS-TC-H30-ARKF	PURE-INS (Tunnel)
E	10.043	10.204	10.737	6.127	33.188
N	11.878	8.738	5.452	5.880	21.700
H	15.555	13.434	12.042	8.492	39.653
Max (H)	N/A	40.540	47.504	21.918	88.834

## Data Availability

No new data were created or analyzed in this study. Data sharing is not applicable to this article.
